# Different characteristics of heart failure due to pump failure and bradyarrhythmia

**DOI:** 10.1007/s12574-014-0235-z

**Published:** 2014-12-06

**Authors:** Mai Iwataki, Yun-Jeong Kim, Byung-Joo Sun, Jeong-Yoon Jang, Masaaki Takeuchi, Shota Fukuda, Kyoko Otani, Hidetoshi Yoshitani, Hisaharu Ohe, Ritsuko Kohno, Yasushi Oginosawa, Haruhiko Abe, Robert A. Levine, Jae-Kwan Song, Yutaka Otsuji

**Affiliations:** 1 Second Department of Internal Medicine, University of Occupational and Environmental Health, School of Medicine, 1-1 Iseigaoka, Yahatanishi-ku, Kitakyushu, 807-8555 Japan; 2 Department of Heart Rhythm Management, University of Occupational and Environmental Health, School of Medicine, Kitakyushu, Japan; 3 Echocardiography Laboratory, Asan Medical Center, Seoul, Korea; 4 Cardiac Ultrasound Laboratory, Massachusetts General Hospital, Boston, MA USA

**Keywords:** Echocardiography, Heart failure, Bradyarrhythmia, Left ventricular pump failure

## Abstract

**Background:**

Heart failure (HF) can be caused by left ventricular (LV) pump failure as well as by bradyarrhythmias. Hemodynamic differences between HF by LV pump failure and that by bradyarrhythmia have not been fully investigated. We hypothesized that HF by LV pump failure could be associated with both reduced cardiac output (CO) and increased LV filling pressure due to associated LV diastolic dysfunction, whereas HF by bradyarrhythmia could be associated with reduced CO but only modestly increased LV filling pressure due to the absence of LV diastolic dysfunction.

**Methods:**

In 39 patients with HF by LV pump failure (LV ejection fraction <35 %), 24 with HF by bradyarrhythmia, and 22 normal controls, LV volume, ejection fraction, stroke volume, left atrial volume, and early diastolic mitral valve flow to tissue annular velocity ratio (E/E′) were measured by echocardiography.

**Results:**

Compared to patients with HF by LV pump failure, those with HF by bradyarrhythmia had significantly lower heart rates, less LV dilatation, preserved LV ejection fraction, preserved stroke volume, similarly reduced cardiac index (1.8 ± 0.4 vs. 1.6 ± 0.4 L/min/m^2^, n.s.), preserved LV diastolic function (E′) (4.4 ± 2.1 vs. 7.1 ± 2.9 cm/s, *p* < 0.001), less dilated end-systolic LA volume, and preserved E/E′ (24 ± 10 vs. 13 ± 7, *p* < 0.001).

**Conclusions:**

HF by LV pump failure is characterized by both significantly reduced CO and increased LV filling pressure, whereas HF by bradyarrhythmia is characterized by a similar reduction in CO but only modestly increased LV filling pressure.

## Introduction

Heart failure (HF) is a major cause of mortality and poor quality of life [1, 2]. HF can be caused by both left ventricular (LV) pump failure as well as by bradyarrhythmias without LV pump failure [3, 4]. Because LV pump failure and bradyarrhythmia differ considerably, it is expected that the hemodynamic characteristics of HF by pump failure and bradyarrhythmia similarly differ. HF due to bradyarrhythmia is generally considered to cause only modestly increased LV filling pressure, while significantly increased LV filling pressure frequently accompanies pump failure. However, hemodynamic differences between HF due to LV pump failure and that due to bradyarrhythmia have not been fully investigated.

LV pump failure with both systolic and diastolic dysfunction is known to cause both reduced cardiac output (CO) and elevated LV filling pressures or lung congestion [4, 5]. Bradyarrhythmia can also cause reduced CO [6, 7]. However, elevated LV filling pressures or lung congestion related to bradyarrhythmia may not be prominent due to the absence of LV diastolic dysfunction. We, therefore, hypothesized that HF due to LV pump failure could be associated with both reduced CO and increased LV filling pressure, whereas HF by bradyarrhythmia could be associated with reduced CO but only modestly increased LV filling pressure. The purpose of this study is to test this hypothesis by evaluating CO and LV filling pressures using quantitative 2-dimensional and Doppler echocardiography in patients with HF due to LV pump failure and those with HF due to bradyarrhythmia.

## Methods

### Subjects

Subjects consisted of 3 groups meeting the following criteria: (1) 39 consecutive patients who underwent echocardiography due to HF symptoms (dyspnea or fatigue) associated with LV pump failure, defined by an LV ejection fraction <35 % without other structural heart disease; (2) 24 consecutive patients admitted to the hospital due to HF symptoms associated with bradyarrhythmia (sinus bradycardia or atrioventricular block with QRS rate <50 beats/min) but without other structural heart disease or hypothyroidism; and (3) 22 age- and gender-matched healthy controls. The etiology of HF in the bradyarrhythmia group was confirmed by the disappearance of HF symptoms after pacemaker implantation. We collected other data including elements from the present and past history, reports of other blood tests, and interpretations of chest X-rays [8]. None of the healthy controls had hypertension, diabetes, hypercholesterolemia, and/or cardiovascular disease. Using an echocardiographic database, patients meeting the study criteria were retrospectively enrolled in the study. Informed consent was obtained from all patients, and the institutional ethical committee approved the protocol.

### Measurements by echocardiography

Two-dimensional and Doppler echocardiographic examinations were performed in all patients using a commercially available ultrasound machine (iE33, Philips Medical Systems, Andover, MA and Vivid 7, General Electric-Vingmed, Milwaukee, WI). Echocardiography was performed on patients with pump failure at the time of the first hospital visit after the development of HF symptoms. Echocardiography was performed on patients with bradyarrhythmia on the day of admission or the next day. LV end-diastolic volume (LVEDV) and LV end-systolic volume (LVESV) were measured with apical 2- and 4-chamber images using the biplane Simpson’s method [9], and the LV ejection fraction was calculated. End-systolic left atrial (LA) volume was also measured using the biplane Simpson’s method. Interventricular septal diastolic diameter (IVSd) and LV posterior wall dimension at diastole (LVPWd) were measured using two-dimensional measurements in the parasternal long-axis view [10]. Systolic pulmonary arterial pressure (sPAP) was estimated from the peak tricuspid regurgitation (TR) jet according to the simplified Bernoulli equation (sPAP = 4 × *v* ^2^ + right atrial pressure), where *v* is the peak velocity of the TR jet (m/s); right atrial pressure was estimated from the diameter and breath-induced variability of the inferior vena cava [11]. In the parasternal or apical long-axis color Doppler view, mitral regurgitation was quantified by the narrowest jet width or the vena contracta (VC) dimension [12]. Mitral filling flow velocity was recorded with pulsed Doppler echocardiography by placing the sample volume at the mitral leaflet tip in the apical 4-chamber view [13]. The peak Doppler velocities of early (E) and late diastolic flow (A) and the E/A ratio were measured. Tissue Doppler imaging (TDI) of the mitral annulus was obtained in the apical 4-chamber view with a 1.5-mm sample volume placed at the medial mitral annulus. Early diastolic tissue velocity (E′) was measured and the E/E′ ratio calculated [14–18]. Stroke volume (SV) was calculated using a Doppler method. LV outflow tract (LVOT) dimension at the aortic valve annular level was measured in the parasternal long-axis view, and its cross-sectional area was calculated as 3.14 × (LVOT dimension)^2^/4. Velocity time integral (VTI) of LVOT flow was measured using the apical long-axis view. SV was determined as the product of the VTI and the LVOT cross-sectional area. CO was calculated as SV × heart rate [19]. The 2-dimensional speckle tracking analysis was performed using speckle tracking software (EchoPAC PC version 108.1.12: General Electric Medical Systems: QLAB version 7.1: Philips Medical Systems) to obtain LV longitudinal strain measures. For LV longitudinal strain assessment, we used 3 LV apical views, apical 4-chamber, 2-chamber, and long-axis views. After selecting 1 cardiac cycle with optimal image quality, the endocardial border in the end-systolic frame was manually traced. The software automatically generated time-domain LV strain profiles for each of the 6 segments of each view, from which the peak strain was measured. The peak strain at each of the 18 LV segments was determined. The global strain was calculated by averaging the peak strain of the 18 LV segments for each subject [20, 21]. Echocardiographic measurements were averaged over 3 cardiac cycles in patients with LV pump failure and control subjects. The measurements were averaged over 7 cardiac cycles in patients with bradyarrhythmia.

### Statistical analysis

The results were expressed as mean and standard deviation and categorical data as absolute numbers and percentages. Comparisons of continuous variables among the 3 groups were performed by Scheffe’s test. Statistical significance was established at *p* < 0.05. Risk factors and medications groups were compared by the Chi-square test.

## Results

### Clinical characteristics

The clinical characteristics of the study population are presented in Table 1. There were no significant differences in age or gender between patients with LV pump failure and those with bradyarrhythmia. Compared to patients with LV pump failure, those with bradyarrhythmia had a significantly lower incidence of associated coronary artery disease (*p* < 0.001), lower incidence of taking beta-blockers (*p* < 0.05), reduced heart rate (*p* < 0.001), and increased systolic blood pressure (*p* < 0.05). Compared to patients with LV pump failure, those with bradyarrhythmia had a significantly lower incidence of abnormal X-ray, including increased heart size, interstitial edema, intraalveolar edema and pleural effusion, and lower BNP and NT-proBNP levels.

**Table 1:** Clinical characteristics of the study population

	Control (*n* = 22)	LV pump failure (*n* = 39)	Bradyarrhythmia (*n* = 24)	*p*-Value (LV pump failure vs. bradyarrhythmia)
Age (years)	72 ± 6	72 ± 12	77 ± 13	0.27
Male (%)	12 (55)	26 (67)	12 (50)	0.19
NYHA (%)				
II	N/A	6 (15)	18 (75)	<0.001
III	N/A	24 (62)	6 (25)	<0.005
IV	N/A	9 (23)	0 (0)	<0.05
Coronary artery disease (%)	0 (0)	18 (46)	1 (4)	<0.001
History of DM (%)	0 (0)	10 (26)	5 (21)	0.56
Hemoglobin A1c (%)	N/A	6.2 ± 1.0	6.0 ± 0.6	0.33
Hypertension (%)	0 (0)	20 (51)	14 (58)	0.59
Hyperlipidemia (%)	0 (0)	8 (21)	5 (21)	0.98
LDL cholesterol (mg/dl)	N/A	94 ± 40	110 ± 37	0.15
eGFR (ml/min/1.73 m^2^)	N/A	40 ± 29	54 ± 29	0.1
BNP (pg/ml), (*n* = 28)	N/A	1269 ± 1096	238 ± 237	<0.05
NT-proBNP (pg/ml) (*n* = 32)	N/A	20027 ± 14428	2527 ± 3243	<0.001
X-ray				
Increased heart size (%)	N/A	39 (100)	16 (67)	<0.001
Interstitial edema (%)	N/A	38 (97)	8 (33)	<0.001
Intraalveolar edema (%)	N/A	18 (46)	1 (4)	<0.001
Pleural effusion (%)	N/A	25 (64)	4 (17)	<0.001
Medication ACE-I/ARB (%)	0 (0)	17 (44)	12 (50)	0.62
β-blocker (%)	0 (0)	12 (31)	2 (8)	<0.05
Digoxin (%)	0 (0)	0 (0)	1 (4)	0.20
CCB (%)	0 (0)	9 (23)	10 (42)	0.12
Nitrate (%)	0 (0)	0 (0)	0 (0)	N/A
HR (bpm)	60 ± 9	84 ± 17*	39 ± 5*	<0.001
Atrial fibrillation	0/22	3/39	0/24	
Sinus bradycardia	0/22	0/39	8/24	
Atrioventricular block	0/22	0/39	16/26	
Systolic BP (mmHg)	127 ± 8	131 ± 27	148 ± 28*	<0.05
Diastolic BP (mmHg)	73 ± 9	76 ± 18	70 ± 16	0.40

*ACE-I* angiotensin-converting enzyme inhibitor, *ARB* angiotensin receptor blocker, *BNP* brain natriuretic peptide, *BP* blood pressure, *CCB* calcium channel blocker, *DM* diabetes mellitus, *eGFR* estimated glomerular filtrating rate, *HR* heart rate, *LDL* low-density lipoprotein, *NT-proBNP* N-terminal proB-type natriuretic peptide, *NYHA* New York Heart Association

* *p* < 0.05 vs. control

### Hemodynamic similarities and differences between patients with LV pump failure and those with bradyarrhythmia

Compared to controls, LV end-diastolic volume index (LVEDVI) was significantly increased in the LV pump failure groups (*p* < 0.05) and it tended to be increased in the bradyarrhythmia group, but without reaching statistical significance (Table 2). Compared to patients with LV pump failure, those with bradyarrhythmia had lower increase in LVEDVI (*p* < 0.001). Compared to controls, only the LV pump failure group had a significant reduction in the LV ejection fraction by definition (*p* < 0.001). Compared to controls, the SV index was significantly reduced in patients with LV pump failure (*p* < 0.05). The SV index tended to be greater in patients with bradyarrhythmia compared to controls, but without reaching statistical significance. Compared to controls, the CO index was significantly and similarly reduced in both groups (*p* < 0.05), and there was no significant difference between the 2 groups. Compared to patients with LV pump failure, those with bradyarrhythmia had lower increase in TR velocity (*p* < 0.05) and sPAP (*p* < 0.005). The mitral regurgitation (MR) vena contracta dimension was significantly increased in both groups and was significantly less pronounced in patients with bradyarrhythmia (*p* < 0.001). Mitral E velocity and E/A was significantly increased in patients with LV pump failure (*p* < 0.05). E-wave deceleration time (E-DcT) was significantly decreased in patients with LV pump failure (*p* < 0.05). Mitral annular tissue E velocity was significantly reduced only in patients with LV pump failure (*p* < 0.05). Consequently, the early diastolic mitral flow to tissue velocity ratio (E/E′) was significantly increased only in patients with LV pump failure (*p* < 0.001). Compared to controls, both the LV pump failure and bradyarrhythmia groups had significantly increased LA volume indices (*p* < 0.05). Compared to the LV pump failure group, patients with bradyarrhythmia had significantly less pronounced LA dilatation (*p* < 0.05). LV global longitudinal strain was significantly decreased in patients with LV pump failure (*p* < 0.05), while patients with bradyarrhythmia even had increased LV global longitudinal strain compared to controls (*p* < 0.05).

**Table 2:** Comparison of hemodynamics between HF by LV pump failure and HF by bradyarrhythmia

	Control (*n* = 22)	LV pump failure (*n* = 39)	Bradyarrhythmia (*n* = 24)	*p*-Value (LV pump failure vs. bradyarrhythmia)
LVEDVI (ml/m^2^)	56 ± 12	102 ± 31*	63 ± 8	<0.001
LVESVI (ml/m^2^)	20 ± 6	76 ± 26*	21 ± 7	<0.001
LVEF (%)	64 ± 7	26 ± 6*	67 ± 9	<0.001
SVI (ml/m^2^)	38 ± 8	22 ± 7*	43 ± 7	<0.001
CI (L/min/m^2^)	2.1 ± 0.4	1.8 ± 0.4*	1.6 ± 0.4*	0.41
LAVI (ml/m^2^)	23 ± 7	55 ± 25*	42 ± 15*	<0.05
IVSd thickness (mm)	9.5 ± 0.9	9.9 ± 1.7	9.7 ± 1.8	0.93
LVPWd thickness (mm)	9.0 ± 1.0	10.5 ± 1.8*	10 ± 1.5	0.52
Mitral E (cm/s)	67 ± 16	94 ± 30*	84 ± 24	0.33
A (cm/s)	74 ± 15	75 ± 32	81 ± 33	0.74
E/A	0.9 ± 0.2	1.4 ± 0.8*	1.2 ± 0.6	0.39
E-DcT (ms)	271 ± 53	163 ± 59*	256 ± 75	<0.001
TDI E′ (cm/s)	6.3 ± 1.6	4.4 ± 2.1*	7.1 ± 2.9	<0.001
E/E′	11 ± 4	24 ± 10*	13 ± 7	<0.001
MR vena contracta (mm)	0 ± 0	3.4 ± 1.4*	1.9 ± 1.0*	<0.001
TR velocity (m/s)	N/A	3.0 ± 0.4	2.7 ± 0.4	<0.05
Systolic PAP (mmHg)	N/A	49 ± 12	40 ± 11	<0.005
LV global longitudinal strain (%)	−15.6 ± 2.5	−7.5 ± 2.2*	−19.1 ± 5.1*	<0.001

*A* late diastolic flow velocity, *CI* cardiac index, *E* early diastolic flow velocity, *E/A* ratio of early transmitral flow velocity to atrial flow velocity, *E-DcT* E-wave deceleration time, *HF* heart failure, *IVSd* interventricular septal diastolic diameter, *LAVI* left atrial volume index, *LVEDVI* left ventricular end-diastolic volume index, *LVEF* left ventricular ejection fraction, *LVESVI* left ventricular end-systolic volume index, *LVPWd* left ventricular posterior wall dimension at diastole, *MR* mitral regurgitation, *PAP* pulmonary arterial pressure, *TDI* tissue Doppler imaging, *TR* tricuspid regurgitation, *SVI* stroke volume index

* *p* < 0.05 vs. control

### Representative images of a patient with LV pump failure and another patient with bradyarrhythmia

**Fig. 1:**
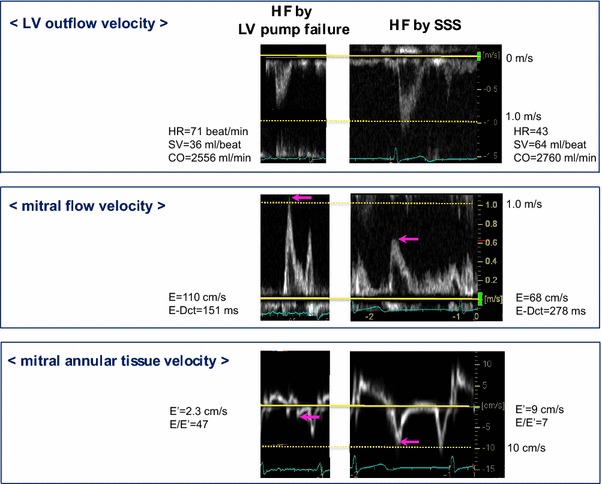
Representative images in a patient with left ventricular (LV) pump failure and another patient with bradyarrhythmia. *Upper panels* reduction in stroke volume (SV) in LV pump failure is evident by reduced LV outflow tract velocity, whereas SV is preserved in the patient with bradyarrhythmia. Due to the difference in heart rate, cardiac output is similarly reduced in both patients. *Middle and lower panels* increase in early diastolic mitral flow E velocity is less pronounced in the patient with bradyarrhythmia. Early diastolic mitral annular tissue E′ velocity is reduced in LV pump failure, whereas it is preserved in the patient with bradyarrhythmia, resulting in increased E/E′ only in the patient with LV pump failure

As shown in Fig. 1 (upper panels), SV is considerably reduced in this patient with LV pump failure, whereas it was preserved in the patient with bradyarrhythmia. Due to the difference in HR, CO is similarly reduced in both patients. As shown in the middle panels, the increase in early diastolic mitral flow E velocity tended to be less pronounced in the patient with bradyarrhythmia. As shown in the lower panels, early diastolic mitral annular E′ velocity was considerably reduced in the patient with LV pump failure, whereas it was preserved in the other patient with bradyarrhythmia. Consequently, E/E′ was considerably increased only in the patient with LV pump failure.

### Comparison between patients with sinus bradycardia and those with atrioventricular block

Table 3 summarizes the data of patients with sinus bradycardia and those with atrioventricular block. Patients with atrioventricular block had increased systolic BP and LV wall thickness. HF symptoms tended to be more severe and pulmonary artery systolic pressure was significantly elevated in patients with atrioventricular block, while LV diastolic functional indices, including E/E′, were comparable between the 2 groups.

**Table 3:** Comparison of hemodynamics between HF by sinus bradycardia and HF by atrioventricular block

	Sinus bradycardia (*n* = 8)	Atrioventricular block (*n* = 16)	*p*-Value
Age	71 ± 13	79 ± 12	0.15
Male (%)	3 (38)	9 (56)	0.39
NYHA			
II	7/8	11/16	0.32
III	1/8	5/16	0.32
Hypertension (%)	3/8	11/16	0.14
HR (bpm)	37 ± 6	40 ± 5	0.3
Systolic BP (mmHg)	132 ± 19	157 ± 28	<0.05
Diastolic BP (mmHg)	64 ± 16	73 ± 15	0.18
LVEDVI (ml/m^2^)	61 ± 8	63 ± 8	0.46
LVESVI (ml/m^2^)	19 ± 6	22 ± 7	0.38
LVEF (%)	69 ± 9	66 ± 10	0.55
SVI (ml/m^2^)	42 ± 4	44 ± 8	0.51
CI (L/min/m^2^)	1.6 ± 0.4	1.6 ± 0.3	0.75
LAVI (ml/m^2^)	38 ± 13	44 ± 17	0.4
IVSd thickness (mm)	8.6 ± 0.8	10.3 ± 1.9	<0.05
LVPWd thickness (mm)	9.2 ± 0.5	10.5 ± 1.6	<0.05
Mitral E (cm/s)	81 ± 20	86 ± 26	0.63
A (cm/s)	65 ± 20	89 ± 34	0.08
E/A	1.3 ± 0.5	1.1 ± 0.6	0.34
E-DcT (ms)	251 ± 77	258 ± 76	0.84
TDI E′ (cm/s)	6.6 ± 2.0	7.3 ± 3.4	0.62
E/E′	13 ± 5	13 ± 8	1.0
MR vena contracta (mm)	2.3 ± 0.5	1.7 ± 1.1	0.18
TR velocity (m/s)	2.2 ± 0.9	2.8 ± 0.5	<0.05
Systolic PAP (mmHg)	31 ± 13	42 ± 12	<0.05
LV global longitudinal strain (%)	−16.8 ± 3.2	−21.6 ± 3.8	<0.05

## Discussion

This study has demonstrated characteristic similarities and differences between patients with HF due to LV pump failure and those with HF due to bradyarrhythmia. These 2 groups of patients had similar reductions in CO. However, detailed characteristics were different. Patients with LV pump failure had reduced SV with increased heart rate, whereas those with bradyarrhythmia even had an augmented SV with reduced heart rate. Increased heart rate in LV pump failure and augmented SV in bradyarrhythmia may be secondary and compensatory. Consequently, these 2 groups had similarly reduced CO. LV dilatation was more prominent in patients with pump failure compared to those with bradyarrhythmia. This finding suggests that LV dilatation in pump failure can be primary, whereas that in bradyarrhythmia is secondary. Regarding LV filling pressure or lung congestion, these 2 groups showed considerable differences. Patients with LV pump failure seemed to demonstrate a significant increase in LV filling pressure or lung congestion, as suggested by significant increases in mitral flow E velocity, E/E′, and LA volume. In contrast, these changes were not significant or only modest in patients with bradyarrhythmia. These differences can be explained by the presence or absence of LV diastolic dysfunction in patients with LV pump failure and those with bradyarrhythmia, as expressed by reduced or preserved mitral annular tissue E′ velocity, respectively. Therefore, this study has demonstrated hemodynamic similarities and differences between HF due to LV pump failure and that due to bradyarrhythmia.

Regarding differences between sinus bradycardia and atrioventricular block, patients with atrioventricular block had significantly increased systolic BP and LV wall thickness. This could be explained by the tendency of higher ages in patients with atrioventricular block. Patients with atrioventricular block had similarly reduced CO and similarly preserved or even augmented stroke volume. In contrast, patients with atrioventricular block tended to have greater HF symptoms and increased pulmonary artery pressure, while LV diastolic functional indices, including E/E′, were comparable between the 2 groups. In addition to reduced QRS rate, the development of P-waves in inadequate phase, such as ventricular systole, may develop a greater increase in early or mid-systolic LA pressure, which may not be expressed by early diastolic E/E′.

### Relation to previous studies

Both LV pump failure and bradyarrhythmia are known to cause HF with reduced CO and/or increased LV filling pressure [3, 4, 6]. However, to date, no study has compared the degrees of CO reduction and lung congestion or increased LV filling pressure between these 2 etiologic groups. Therefore, characteristic differences in HF due to LV pump failure and HF due to bradyarrhythmia have not been clearly investigated. Our study has demonstrated similarities and differences between HF due to the 2 etiologies. Mullens et al. [22] reported a reduced CO index (2.0 ± 0.7 L/min/m^2^) with considerably increased LV filling pressures (LV end-diastolic pressure of 21 ± 6 mmHg) in patients with LV pump failure. Stack et al. [7] demonstrated only a mild increase in pulmonary artery diastolic pressure of 13 mmHg and a considerably reduced CO index of 1.69 L/min/m^2^ in patients with bradyarrhythmia. These studies support the results of the present study. Our study has also demonstrated the association or dissociation of LV diastolic dysfunction in patients with LV pump failure and those with bradyarrhythmia.

### Clinical implications

This study has demonstrated that both reduced CO and increased LV filling pressures can be prominent in patients with LV pump failure, whereas increased LV filling pressures is only modest in patients with HF due to bradyarrhythmia. Therefore, a marked increase in LV filling pressures in patients with bradyarrhythmia may suggest associated LV pump failure or other causes of elevated LV filling pressures. It may also be useful to expect that pacing to increase the heart rate in patients with bradyarrhythmia may markedly increase CO, whereas it may not result in a satisfactory improvement in elevated LV filling pressures. In this study, indices of LV mechanical function, including ejection fraction, SV, and mitral annular tissue E′ velocity, were all preserved or even augmented in patients with bradyarrhythmia. These findings suggest compensatory LV mechanical adaptation to reduced heart rate. If indices of LV mechanical function were reduced in patients with bradyarrhythmia, this finding may suggest the presence of associated LV structural disease other than bradyarrhythmia.

### Limitations

Although echocardiography is largely established as a modality to accurately measure SV or CO [19, 25], echocardiographic evaluation of LV filling pressure or lung congestion remains controversial [22–24, 26, 27]. In this study, LV filling pressure was non-invasively evaluated by echocardiography and was not directly measured by catheterization. This study was not prospective. The patients were retrospectively enrolled from an existing echocardiographic database. The timing of echocardiographic examination was not necessarily at the time of peak HF symptoms. Often, the patients were treated with diuretics and other medications prior to echocardiography examination, and HF symptoms were often attenuated at the time of echocardiography. The use of medication was also not controlled. In general, the duration of bradycardia was not clarified. In cases of associated LV pump failure and bradyarrhythmia, echocardiographic evaluation of LV functional indices of both systole and diastole can be considered for better evaluation. However, such patients were not investigated. Differences in hemodynamics between the 2 major etiologies of bradyarrhythmia (sinus bradycardia and atrioventricular block) are especially interesting. However, such differences were not significantly different potentially due to the limited number of patients.

## Conclusions

These results suggest that heart failure (HF) due to left ventricular (LV) pump failure is characterized by both a significant reduction in cardiac output (CO) and increased LV filling pressure, whereas HF by bradyarrhythmia is characterized by a similar reduction in cardiac output but only a modest increase in LV filling pressure.

## Abbreviations

CO: Cardiac output
E: Early diastolic flow velocity
E′: Early diastolic tissue velocity
EF: Ejection fraction
HF: Heart failure
LA: Left atrium/atrial
LV: Left ventricle/ventricular
TDI: Tissue Doppler imaging
